# The impact of the COVID-19 pandemic on faculty in nursing education: a scoping review

**DOI:** 10.1186/s12912-025-03550-7

**Published:** 2025-07-08

**Authors:** Ina Thon Aamodt, Elisabeth Østensen, Irene Valaker, Kristin Valen, Gøril Tvedten Jorem, Anne Kristin Snibsøer

**Affiliations:** 1 Department of Nursing and Health Promotion, Faculty of Health Science, Oslo Metropolitan University, Pb 4 St. Olavs Plass, Oslo, Norway; 2 Department of Postgraduate Studies, Lovisenberg Diaconal University College, Oslo, Norway; 3 Department of Health and Caring Sciences, Faculty of Health and Social Sciences, Western Norway University of Applied Sciences, Førde, Norway; 4 Department of Health and Caring Sciences, Faculty of Health and Social Sciences, Western Norway University of Applied Sciences, Haugesund, Norway; 5 Library, Western Norway University of Applied Sciences, Bergen, Norway; 6 Department of Health and Caring Sciences, Faculty of Health and Social Sciences, Western Norway University of Applied Sciences, Bergen, Norway

**Keywords:** Nursing faculty, Nursing education, COVID-19, Scoping review

## Abstract

**Background:**

The COVID-19 pandemic influenced the delivery of nursing education worldwide. The objective of this scoping review was to map the existing research literature on how the COVID-19 pandemic impacted faculty in nursing education professionally and personally.

**Methods:**

The framework of Joanna Briggs Institute Manual for Evidence Synthesis guided the development of this scoping review. Publications were searched for in the following databases: Medline, Embase, CINAHL, ERIC, Epistemonikos, Cochrane, Web of Science, Scopus, in addition to Teacher Reference Center, and Google Scholar. Frequency counts were registered to record the characteristics of sources of evidence. Frequency counts and summarized descriptions were then used to understand how nursing faculty was affected professionally and personally.

**Results:**

After screening a total of 8525 publications, 34 articles were found eligible for inclusion. The publications originated worldwide with the majority from North America and Asia. Included studies made use of qualitative, quantitative, and mixed methods to illuminate, experiences and descriptions of the challenges facing nursing faculty in academia during the COVID-19 pandemic. The nursing faculty was professionally impacted by COVID-19 pandemic as it led to an online working environment, challenging workloads, working hours, teaching methods, and less focus on research. The personal impact of the COVID-19 pandemic on faculty members was related to physical, emotional and social aspects.

**Conclusions:**

This scoping review mapped the literature on how the COVID-19 pandemic impacted faculty in nursing education professionally and personally. Nursing education was unprepared for such a crisis. Our findings call for future studies focusing on long term online working environment for nursing faculty is encouraged as it will benefit nursing education.

**Supplementary Information:**

The online version contains supplementary material available at 10.1186/s12912-025-03550-7.

## Background

Nursing education is an essential aspect of global sustainable healthcare provision, with a projected worldwide need-based shortfall of 4.5 million nurses by 2030 [1, 2]. The report titled State of the World’s Nursing 2020 encourages governments and relevant stakeholders to promote nursing education by investing in nursing faculty, infrastructure and students [1]. However, nursing education faces challenges in recruiting and retaining nursing faculty due to low compensation, workload and many faculty retiring [3].

The global COVID-19 crisis abruptly impacted nursing faculty as they had to redesign nursing courses to online teaching and learning to enhance the safety of nursing faculty and students.

In a previous scoping review, Amankwaa et al. [4] found that interventions in nursing education developed in response to ongoing COVID-19, were mainly online/virtual teaching as an alternative to face-to-face teaching [4]. Moreover, the authors report of developing online/virtual teaching support programs and simulation as an alternative to clinical placement and support programs [4]. Findings from another scoping review examining perception and self-efficacy of nurse educators’ response to COVID-19 pandemic, present challenges with online education [5]. In addition, an uncertainty in the role as educator during a pandemic, technical issues and an impact on the well-being of faculty was described [5]. Also, a systematic review and meta-synthesis conducted among undergraduate students and nursing faculty revealed mixed experiences of remote and online education during the COVID-19 pandemic [6]. In this review more experiences were described by undergraduate students than by faculty members [6]. These previous reviews included nursing educators in academia, clinical instructors and nursing students and their searches were limited to 2022 [4–6]. None of the reviews had focused exclusively on nursing faculty in academia, a focus that is encouraged in future studies [7].

Thus, the objective of this scoping review was to map the existing research literature on how the COVID-19 pandemic impacted nursing faculty at all educational levels in academia professionally and personally.

## Methods

This scoping review used the framework initially designed by Arksey and O’Malley [8], enhanced by Levac et al. [9], and further developed by Peters et al. [10].

To guide the development of the review, we adopted the following stages of the JBI Manual for Evidence Synthesis [10]: (1) defining the review question, (2) developing inclusion and exclusion criteria, (3) describing the planned approach to evidence searching, selection, data extraction and presentation of evidence, (4) searching for the evidence, (5) selecting the evidence, (6) extracting the evidence, (7) analyzing the evidence, (8) presenting the results and (9) summarizing the evidence in relation to the review objective making conclusions and implications of findings.

A protocol was developed, using the template from Joanna Briggs Institute (JBI) resources. The protocol included pre-defined objectives, methods, and reporting of the review [10]. The protocol was not registered in a database. For reporting of the review, the checklist of the Preferred Reporting Items for Systematic Reviews and Meta-Analysis Extension for Scoping Reviews (PRISMA ScR) [11] was used (Supplement 1).

### Review questions

The following two review questions were formulated.

1. How was nursing faculty professionally affected by the COVID-19 pandemic?
2. How was nursing faculty personally affected by the COVID-19 pandemic?

### Eligibility criteria

The eligibility criteria are presented in Table 1 with population, concept and context and sources of evidence (Table 1).

#### Population

Studies eligible for inclusion were studies that included nursing faculty who taught nursing students at the bachelor/undergraduate, the master/postgraduate level and/or PhD level during the COVID-19 pandemic. Empirical studies that reported on nursing faculty teaching healthcare professionals or students in other health-related professions were excluded as were studies reporting on clinical instructors exclusively teaching in the clinical setting. Studies with mixed populations (e.g., students, faculty and clinicians) were included when findings from nursing faculty in academia were reported independently from other participants. Publications that examined teaching not specifically undertaken due to the COVID-19 pandemic were excluded.

#### Concept

The questions addressed in this scoping review were how the COVID-19 pandemic impacted nursing faculty professionally and personally. The phenomena of interest were the professional impact that included aspects related to teaching strategies, and research, working conditions in terms of environment, support, resources, workload and working hours. The other phenomena of interest were the personal impact that included psychological, physical and social aspects. Publications that solely evaluated teaching programs developed and tested during Covid-19 were excluded.

#### Context

We included studies that examined nursing faculty working in educational institutions teaching nursing such as colleges and universities. Publications exclusively undertaken in a clinical setting were excluded. No limitations were applied to geographical location.

#### Sources of evidence

The review included all types of study designs (e.g., quantitative, qualitative and mixed methods) published in English. Editorials, letters, opinion papers, conference proceedings, all types of reviews (systematic review, scoping, integrative, umbrella and narrative) and studies published in other languages than English was excluded.

**Table 1:** Overview of eligibility criteria

	Inclusion criteria	Exclusion criteria
Population	Nursing faculty teaching nursing during the COVID-19 pandemic Studies reporting independent results from nursing faculty in academia	Faculty teaching healthcare professionals or students in other health-related professions Instructors exclusively teaching in the clinical setting Teaching not specifically undertaken due to the COVID-19 pandemic
Concept	Professional and personal impact of the COVID-19 pandemic	Evaluation of teaching programs
Context	Educational institutions (colleges and universities) All educational levels All geographical locations	Clinical setting
Sources of evidence	All types of study designs (e.g. quantitative, qualitative, mixed methods) Published in English	Editorials, letters, opinion papers, conference proceedings, all types of review studies (systematic, scoping, integrative, umbrella, and narrative) Non-English publications

### Search strategy

As recommended by JBI resources [10] we conducted a three-step search strategy. First an initial limited search of Medline and CINAHL was performed to identify articles on nursing faculty during COVID-19. The keywords contained in the titles and abstracts of relevant retrieved articles, and the index terms used to describe these studies were then used to develop a full search strategy for Ovid Medline (Appendix 1). The development of the search string was performed by one of the authors (AKS) and an academic librarian (GTJ), and it was peer-reviewed by another academic librarian. The search strategy focused on the two major aspects of our topic: COVID-19 and nursing education.

A second search was performed in the following databases: Medline, Embase, CINAHL, ERIC, Epistemonikos, Cochrane, Web of Science and Scopus. In this search, all identified keywords and index terms were adopted for each included database. In addition, the Teacher Reference Center and Google Scholar were searched. The primary search was performed in June 2022 (Appendix 2), and an updated search was conducted in September 2023 (Appendix 3).

In the third search, we looked for citation and screened reference lists for additional studies. As the COVID-19 virus first appeared during autumn of 2019, the search was limited to literature from August 2019 onwards.

### Screening and selecting the sources of evidence

All identified citations were collated and uploaded to the reference management software EndNote 20. Duplicate removal and screening was undertaken in the Rayyan online tool [12]. Prior to the screening of titles and abstracts, we performed a pilot test of 50 random publications. In this process, all researchers used the eligibility criteria to screen the titles and abstracts independently. Discrepancies were discussed and modifications to the eligibility criteria were made to ensure agreement among the researchers.

**Fig. 1:**
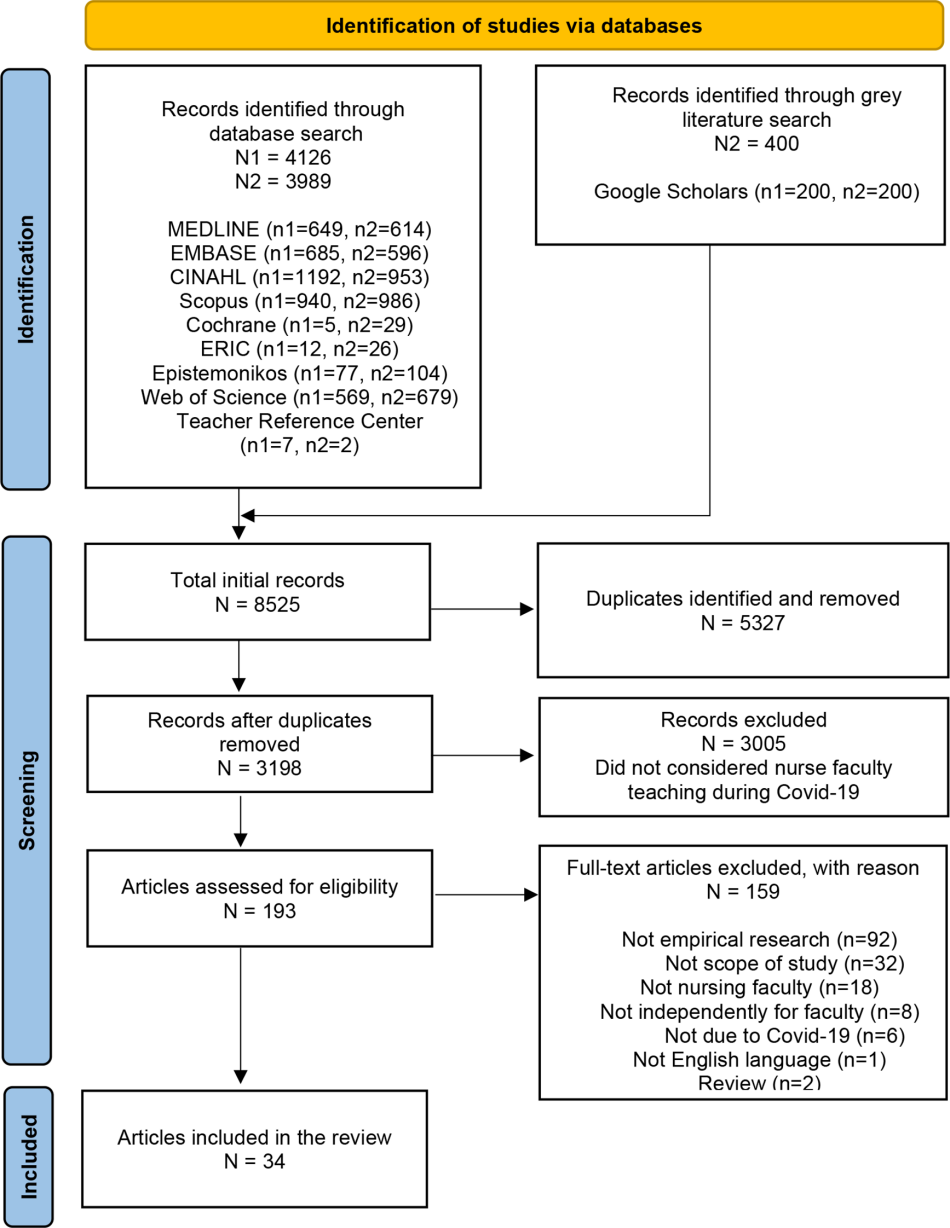
PRISMA-ScR flow diagram

After the pilot test, two pairs of researchers (ITA and EØ, AKS and IV) independently screened titles and abstracts against the eligibility criteria. Disagreements were resolved through consultation and discussion with a fifth researcher (KV). All potentially relevant sources were retrieved in full text. Further, the same pairs of researchers screened the selected full-text citations in detail by using the inclusion criteria. The exclusion of sources in full text was mainly related to the evidence source and scope of the study (Fig. 1). The search results and the study inclusion process are presented in the PRISMA-ScR flow diagram, adapted from Page et al. [13].

### Data extraction

To facilitate systematic charting of data, we developed a data extraction sheet based on the template by JBI resources [10]. The extraction sheet was piloted by all researchers on 5 publications. Some modifications were made to ensure applicability to the objective of this scoping review. Further, two pairs of researchers (ITA and AKS, and EØ and KV) extracted data independently. Any disagreements between them were resolved through discussion, or by the decision of a third researcher (IV).

The following information was extracted:

- Author(s), year of publication, journal.
- Aim(s) of the study.
- Study population (nursing faculty in academia).
- Context (university/college, educational level, country of origin).
- Methodology (design, data collection, data collection period).
- Professional impact of COVID-19 (teaching strategies, research, working conditions, environment, support, resources, workload, and working hours).
- Personal impact of COVID-19 (psychological, physical, social aspects).

### Data analysis

Frequency counts were conducted to register the characteristics of sources of evidence [10]. Further, we summarized descriptions of the concept [14] professional and personal impact of the COVID-19 pandemic on nursing faculty.

## Results

### Selection of sources of evidence

A total of 8525 studies were identified in the first (n1 = 4336) and the updated (n2 = 4189) database search (Fig. 1). After removing duplicates, 3198 records were screened for title and abstract. Of these, 3005 were excluded as they did not relate to the nursing faculty during COVID-19. The remaining 193 records were subject to full-text screening, and 159 records were excluded with reasons (Fig. 1). Thirty-four studies met the eligibility criteria and were included in the review and are presented in Table 3.

### Characteristics of sources of evidence

The included studies were conducted at universities and colleges with nursing programs in countries worldwide. Most studies were from North America. (Table 2).

**Table 2:** Overview of continents, the number (n) of countries and the reference to the studies

Continent	*n* = 34	Country (reference)
Africa	2	Namibia [15], South Africa [16]
North America	14	Canada [17–19], United States [20–30]
South America	2	Brazil [31, 32],
Asia	9	Cambodia, Hong Kong, Indonesia, Japan, Laos, Malaysia, Philippines, Singapore, Thailand, Vietnam [33] India [34, 35], Indonesia [36], Iran [37], Japan [38], Jordan [39] Nepal [40], Palestina [41]
Europe	4	Norway [42], Spain [43], Turkey [44, 45]
Oceania	1	Australia [46]
More than 2 continents	2	Australia and United Kingdom [47] Kenya, Canada, USA, Ireland, United Kingdom, Australia [48]

The included studies used quantitative (*n* = 13), qualitative (*n* = 19), and mixed methods (*n* = 2). All the quantitative studies were cross-sectional, with the online survey as the preferred data collection method. The applied measurement tools were pre-existing questionnaires [15–20], questionnaires developed for the current study in question [21–24], or a combination of the two [25–27]. Of the studies that developed specific questionnaires for the study (*n* = 4) or a combination of developed and pre-existing questionnaires (*n* = 3), face or content validity was reported in three publications [21, 26, 27]. The mixed method studies used surveys with pre-existing questionnaires, presenting open-ended questions as themes [20, 28].

Eleven of the nineteen qualitative studies reported a descriptive or exploratory approach [29–39]. A phenomenological approach [40–43], hermeneutic phenomenological [44, 45], grounded theory [46] and an action research approach based on the theoretical framework of Paulo Freire [47] was also found in the included publications. Data were collected in individual interviews [29, 30, 32, 33, 35–39, 41, 42, 44–46] and in focus groups [34, 43]. To reach the informants, researchers used virtual platforms [32, 34, 35, 37, 38, 43, 44, 46, 47], virtual platform or telephone [39, 41, 42], only telephone [45] virtual platform or face-to-face interviews [29, 30, 33, 36]. Three studies described data collection in face-to-face interviews [29, 33, 36]. Other data collection methods were open-ended questionnaires [15, 16, 21, 25, 28, 31, 48], self-recorded videos and self-reflective notes [40].

Specific data collection periods were reported in most publications but were lacking in six (Table 3). In these publications, the missing dates were described as “ongoing pandemic” [19, 22, 48], “teaching experiences during the COVID-19 pandemic” [30] and “nationwide lockdown” [24, 43].

In most publications the nursing faculty were engaged in bachelor programs. Twelve studies reported nursing faculty working at diverse educational levels [16, 21, 22, 25, 28, 30, 32, 34, 35, 37, 38, 45]. Six studies did not report on educational level. In these publications nursing faculty described working at universities with nursing programs [18, 23, 27], nursing colleges [19, 24] or working as nursing educators [39].

The frequency and summarized descriptions of the professional impact of COVID-19 pandemic are described in the following as: Working environment, Teaching and research, Workload and working hours. The personal impact of COVID-19 pandemic is described in the following as: Psychological, physical, and social aspect.

**Table 3:** Data extraction from the included studies *n* = 34

Author, year	Aim	Journal	Methods	Data collection	Sample	Context	Main Concept
Atout et al., 2022	To explore the experience of online education during COVID-19 from the perspective of nurse educators, postgraduate students and undergraduate students.	Nursing Forum	Qualitative design Focus group interviews	Sept-Oct 2020	Nurse educators (*n* = 14) Postgraduate students (*n* = 9) Undergraduate students (*n* = 30)	Universities (*n* = 2), Bachelor and Postgraduate level Occupied Palestinian Territory	Professional impact of the Covid-19 pandemic: working environment, teaching.
Brown et al., 2022	To explore the experiences and perceptions of academics when teaching university-based healthcare programs during “work from home” initiative.	Nurse Education Today	Qualitative design Individual interviews	May-July 2020	Health academics (nurses, midwives and paramedics) (*n* = 34)	Universities Australia (*n* = 6) United Kingdom (*n* = 2) Bachelor and postgraduate level	Professional impact of the Covid-19 pandemic: working environment, workload, working hours, teaching. Personal impact of the Covid-19 pandemic: psychological, physical and social.
Christoffers et al., 2023)	How nursing faculty navigated the challenges in an online nursing program and how they were able to support their students during the CCOVID-19 global pandemic.	Nursing Clinics of North America	Qualitative design Individual interviews	April 2022	Nurse faculty members (*n* = 7)	Online RN-to-BSN nursing program at a 4-year institution in the Upper Midwest, United States	Professional impact of the Covid-19 pandemic: workload, teaching. Personal impact of the Covid-19 pandemic: psychological and social.
Christoffers et al., 2023	To explore the transformation of perspectives that nursing faculty teaching in online RN-BSN programs experienced.	Journal of Transformative Education	Qualitative design Individual interviews	March-Oct 2022	Nurse faculty members (*n* = 10)	Online RN-to-BSN nursing program at a 4-year institution in the upper-Midwest, United States	Professional impact of the Covid-19 pandemic: teaching. Personal impact of the Covid-19 pandemic: psychological and social
Cobbett et al., 2022	To investigate student and faculty experiences during swift pedagogical shift to immersion in the online environment during a global pandemic. (Part 1)	Quality Advancement in Nursing Education	Cross-sectional design, online survey	July-Sept 2020	Nurse faculty (*n* = 30) students (*n* = 193)	Universities (*n* = 3), Bachelor level Canada	Professional impact of the Covid-19 pandemic: working environment, workload, teaching. Personal impact of the Covid-19 pandemic: psychological.
Cobbett et al., 2022	To explore the experience of nursing students and faculty when learning and teaching in the fully online environment during COVID-19 pandemic. (Part 2)	Quality Advancement in Nursing Education	Qualitative design Open-ended questions in survey	July-Sept 2020	Nurse faculty (*n* = 38) undergraduate nursing students (*n* = 195)	Universities (*n* = 3), Bachelor level Canada	Professional impact of the Covid-19 pandemic: workload, teaching. Personal impact of the Covid-19 pandemic: psychological and social.
de Souza, 2021	To reflect on how undergraduate nursing teachers experience the activities of their work process in the COVID-19 pandemic context.	Cogitare Enfermagem	Qualitative design Action research	Aug 2020	Nurse teachers (*n* = 20)	Universities, Bachelor level southern Brazil	Professional and personal impact of the Covid-19 pandemic: working environment, workload, working hours, teaching. Personal impact of the Covid-19 pandemic: psychological and physical.
Farber et al., 2023	To examine life balance and professional quality of life among nurse faculty in 2021 during the first year of the COVID-19 pandemic and to describe the challenges of delivering virtual learning experiences.	Journal of Professional Nursing	Cross-sectional design, online survey	March-April 2021	Nurse faculty (*n* = 216)	Nursing programs (*n* = 10) offering baccalaureate or post-baccalaureate degree, USA	Professional and personal impact of the Covid-19 pandemic: workload, teaching. Personal impact of the Covid-19 pandemic: psychological.
Gazza, 2022	To uncover the experience of being full-time academic nurse educators in baccalaureate or higher degree nursing programs during the COVID-19 pandemic.	Nursing Education Perspectives	Qualitative design Individual interviews	April 2020	Nurse educators (*n* = 14)	Bachelor, master`s or doctoral level of nursing program in North Carolina, USA	Professional impact of the Covid-19 pandemic: working environment, teaching.
Gutierres-Ruivo et al., 2023	To analyze fatigue among nurse educators in public universities when implementing Emergency Remote Teaching during the Covid-19 pandemic.	Texto & Contexto Enfermagem	Cross-sectional design, online survey	July-Nov 2021	Nurse educators (*n* = 318)	Federal and state universities, Bachelor level Brazil	Personal impact of the Covid-19 pandemic: psychological and physical.
Iheduru-Anderson, 2021	To explore the experiences of associate degree nursing faculty who transitioned from face -to-face to online teaching during the early months of the COVID-19 pandemic.	Global Qualitative Nursing Research	Qualitative design Individual interviews	May-Aug 2020	Nurse educators (*n* = 41)	Associate degree nursing education level USA	Professional and personal impact of the Covid-19 pandemic: working environment, workload, working hours, teaching. Personal impact of the Covid-19 pandemic: psychological, physical and social.
Ince & Dincer, 2023	To determine the experiences of nurse academicians and their perspectives on possible future pandemics.	European Journal of Clinical and Experimental Medicine	Qualitative design Individual interviews	Sept-Dec 2022	Nurse academicians (*n* = 11)	Bachelor level Turkey	Professional impact of the Covid-19 pandemic: working environment, workload, teaching. Personal impact of the Covid-19 pandemic: psychological, physical and social.
Keener et al., 2021	To examine the relationship of QoL, resilience, and associated factors among nursing faculty during the unprecedented COVID-19 pandemic and subsequent social distancing requirements.	Nurse Educator	Cross-sectional design, online survey	April 2020	Nurse faculty (*n* = 52)	School of Nursing offering baccalaureate, master, and doctoral programs, USA	Professional impact of the Covid-19 pandemic: working environment. Personal impact of the Covid-19 pandemic: psychological and social.
Leigh et al., 2021	To explore the feelings of both students and faculty associated with the transition to remote education.	Teaching and Learning in Nursing	Mixed methods exploratory design, pilot study	In the era of coronavirus pandemic	Nurse faculty (*n* = 31) students (*n* = 363)	Pre-licensure baccalaureate of science nursing (BSN) program, USA	Professional impact of the Covid-19 pandemic: workload. Personal impact of the Covid-19 pandemic: psychological.
Titan Ligita et al. (2022)	To explore nursing lecturers` experience of online teaching during the COVID-19 pandemic.	Health Education	Qualitative design Individual interviews	July-Nov 2020	Nurse lecturers (*n* = 7)	Bachelor level Indonesia	Professional impact of the Covid-19 pandemic: working environment, teaching.
Losa–Iglesias et al., 2022	To assess whether a relationship between factors such as resilience, self-esteem, depression, anxiety, academic stressors, and changes in teaching and learning methods exists and to check whether the pandemic is having an impact on teachers of the nursing degree.	Frontiers in Psychology	Cross-sectional design, online survey	Oct-Nov 2021	Staff nurses and Nurse professors (*n* = 53)	Nursing degree teachers at University Bachelor level, Spain	Personal impact of the Covid-19 pandemic: psychological.
Mokoena-de Beer, 2022	To obtain an in-depth understanding of nurse lectures` experiences with online teaching during COVID-19 pandemic.	Curationis	Qualitative design Individual interviews	During the CVID-19 pandemic	Nurse lectures (*n* = 6)	Undergraduate and postgraduate nursing programs level South Africa	Professional impact of the Covid-19 pandemic: working environment, teaching
Moradi et al., 2022	To explain the challenges experienced by nurse educators and undergraduate nursing students in the sudden shift to asynchronous e-learning during the COVID-19 pandemic.	Nursing and Midwifery Studies	Qualitative design Individual interviews	September to November 2020	Nursing faculty members (*n* = 12) nursing students (*n* = 8)	Bachelor level Iran	Professional impact of the Covid-19 pandemic: working environment, working hours, teaching.
Mudgal et al., 2022	To determine the index of online teaching stress and QOL among nurse educators during COVID-19.	Indian Journal of Psychiatric Nursing	Cross-sectional design, online survey	Dec 2020	Nurse educators (*n* = 162) with a minimum of 3 months’ online teaching experience	Nursing colleges and universities, Bachelor level, India	Personal impact of the Covid-19 pandemic: psychological.
Nabolsi et al., 2021	To explore the lived experience of faculty members who were involved in teaching nursing courses to undergraduate nursing students within the unprecedented online distance learning context which was imposed by the emerging nationwide lockdown due to COVID-19.	Journal of Professional Nursing	Qualitative design Focus group interviews	The country was under complete lockdown and national curfew.	Faculty members (*n* = 15)	Bachelor level, University (*n* = 2), Jordan	Professional impact of the Covid-19 pandemic: working environment, working hours, teaching. Personal impact of the Covid-19 pandemic: psychological.
Nowell, 2021	To gain a more comprehensive understanding of nurse educators` experiences during the pandemic.	Quality Advancement in Nursing Education	Qualitative design Individual interviews	June-July 2020	Nurse educators (*n* = 15)	Educational level is not reported Canada, Australia, Ireland, Kenya United Kingdom, USA	Professional impact of the Covid-19 pandemic: teaching. Personal impact of the Covid-19 pandemic: psychological and social.
Orazietti et al. 2023	To provide an understanding of the lived experiences of nursing faculty teaching clinical courses and nursing students working in Canadian clinical placements settings during the third wave of the COVID-19 pandemic.	Sage Open Nursing	Qualitative design Narratives Self-video, self-written reflection	2020/2021 winter	Nursing students (*n* = 77) Nurse faculty (*n* = 3)	Nursing school, Bachelor level, Canada	Professional and personal impact of the Covid-19 pandemic: workload. Personal impact of the Covid-19 pandemic: psychological, physical and social.
Özberk & Yağcan, 2022	To determine the fundamental factors affecting the perceived stress and organizational commitment of women nurse academicians in the COVID-19 pandemic outbreak.	Health Care for Women International	Cross-sectional design, online survey	Dec 2020-Feb 2021	Female nurse academicians (*n* = 234)	Universities, Nursing degree programs, educational level not reported, Turkey	Professional impact of the Covid-19 pandemic: teaching. Personal impact of the Covid-19 pandemic: psychological and social.
Ridgway, 2022	To describe Australian postgraduate Maternal, Child and Family Health nurse educators` perceptions of COVID-19 impacts on student knowledge of theory and practice, and lessons learned through their responses.	Journal of Professional Nursing	Qualitative design Individual interviews	Dec 2020-Feb 2021	Educators (*n* = 6)	Maternal, Child and Family Health programs (*n* = 3) Postgraduate level, Australia	Professional impact of the Covid-19 pandemic: workload, teaching.
Sacco & Kelly, 2021	To describe nursing faculty experiences during the COVID-19 pandemic response and to compare these experiences based on academic and clinical employment status.	Nursing Education Perspectives	Cross-sectional design, online survey	Summer 2020	Nurse faculty (*n* = 117) teaching at least one didactic course	Nursing programs with undergraduate, graduate, or doctoral nursing programs, USA	Professional and personal impact of the Covid − 19 pandemic: workload, teaching. Personal impact of the Covid-19 pandemic: psychological.
Sessions et al., 2022	To gain a better understanding of the experiences of nurse educators during the COVID-19 crisis.	Teaching and Learning in Nursing	Qualitative design Individual interviews	Feb-April 2021	Nurse educators (*n* = 27)	Nursing programs in Bachelor, Masters and Doctoral level, USA	Professional impact of the Covid-19 pandemic: working environment, workload, working hours, teaching. Personal impact of the Covid-19 pandemic: psychological.
Sessions et al., 2023	To examine the impact of the pandemic on nursing faculties` perceived stress, resilience, and professional quality of life-compassion satisfaction, and whether these contributed to nursing faculty job satisfaction.	Nursing Education Perspectives	Mixed methods design	March-September 2021	Nurse educators (*n* = 278)	Nursing programs across the United States Diploma, associate degree, bachelor, masters` and doctoral programs, USA	Professional impact of the Covid-19 pandemic: working environment, teaching. Personal impact of the Covid-19 pandemic: psychological, social.
Shindjabuluka et al., 2022	To explore nurse educators` experiences of how COVID-19 served as an enabler for the enhancement of online learning and teaching skills with specific emphasis on prospects and challenges.	Health SA Gesondheid	Qualitative design Individual interviews	Aug-Sept 2020	Nurse educators (*n* = 18)	University, A public nursing education, Bachelor level, Namibia	Professional impact of the Covid-19 pandemic: working environment, teaching.
Shorey et al.,2022	To evaluate the impact of the COVID-19 pandemic on nursing students and faculty members` psychosocial well-being as measured by their satisfaction with new learning or teaching modalities, confidence levels, psychosocial well-being, sense of coherence and stress experienced.	Nurse Education Today	Cross-sectional design, online survey	Jan-Aug 2021	Nurse faculty (*n* = 395) Nursing students (*n* = 1897)	Universities (*n* = 13), educational level not reported. Southeast and East Asian Nursing Education and Research Network (SEARN) Cambodia, Hong Kong, Indonesia, Japan, Laos, Malaysia, Philippines, Singapore, Thailand, Vietnam	Personal impact of the Covid-19 pandemic: psychological.
Subedi et al., 2020	To assess the problems faced by students and teachers of Nepal during online classes.	International Journal of Science and Healthcare Research	Cross-sectional design, online survey	At the time of COVID-19 lockdown	Nurse teachers (*n* = 104) students (*n* = 1012)	Nursing colleges (*n* = 13), educational level not reported. Nepal	Professional impact of the Covid-19 pandemic: working environment, workload. Personal impact of the Covid-19 pandemic: psychological and physical.
Svendsen et al., 2023	To attain knowledge about university/college teachers, students, and clinical field instructors’ experiences regarding the digital transition in nursing education.	PLoS ONE	Qualitative design Individual interviews	Spring of 2020	Nurse teachers (*n* = 9) students (*n* = 5) Clinical instructors (*n* = 4)	University (*n* = 2) University college (*n* = 1), bachelor, master’s and doctoral level, Norway	Professional impact of the Covid-19 pandemic: working environment, teaching. Personal impact of the Covid-19 pandemic: social.
Varghese et al., 2022	To identify the level of work stress, to determine the state of general well-being among faculty members and to test the relationship between work stress and general well-being of faculty members during Covid-19 pandemic	The Nursing Journal of India	Cross-sectional design, online survey	During the COVID-19 pandemic	Nurse faculty (*n* = 100)	Selected nursing colleges, educational level not reported, India	Professional impact of the Covid-19 pandemic: working environment, teaching. Personal impact of the Covid-19 pandemic: psychological.
Wilson et al., 2021	Nursing faculty perceptions of the effectiveness of resources, support, and methodologies for online teaching during the COVID-19 pandemic.	Sage Open Nursing	Cross-sectional design, online survey	During the COVID-19 pandemic	Nursing faculty (*n* = 84) who transitioned nursing course to an online format during the COVID-19 pandemic	Nursing colleges and universities (*n* = 10), Bachelor, master and doctor level, USA	Professional impact of the Covid-19 pandemic: teaching
Yoshinaga et al., 2022	To investigate how nursing faculties` perceived time devoted to research changed during the first wave of the pandemic in Japan.	Japan Journal of Nursing Science	Cross-sectional design, online survey	July-Aug 2020	Nursing faculty (*n* = 1023)	Universities with nursing programs, educational level not reported Japan	Professional impact of the Covid-19 pandemic: research

### Working environment

All included studies of the professional impact of the COVID-19 pandemic concerned the shift from a face-to-face to an online working environment. This unexpected and instant shift was required by stay-at-home guidelines to reduce the risk of contracting the COVID-19 virus.

Several studies describe challenges related to the home setting as a working environment. These included available workspace [16, 34, 36], the technical working environment [15, 32, 35, 38, 45], and disruptions due to the presence of family members [16, 19, 36]. In addition, the working environmental guidelines changed during the pandemic [28].

To manage the changed working environment nurse academics used strategies such as physically separating [38] and organizing their household members before starting to work [47]. Faculty also faced technical challenges with unstable electricity and internet access [24, 29, 30, 34, 36, 42] and outdated or lacking hardware and software [33, 41, 43].

### Teaching and research

Teaching practices changed during the pandemic, and studies report on the professional challenges with teaching online. In the shift to online teaching, nursing faculty prepared, redesigned and readapted existing course materials to fit digital platforms [32, 39, 42, 43, 47]. Also, innovative approaches such as multi-media presentations, virtual games [28, 31, 39] and new strategies to include clinical aspects were described. Examples of such approaches were online workshops, available online pre-recorded videos, live video calls, video-demonstration, virtual simulation, multimedia- lectures and labs, and small groups of students with social distancing [15, 30, 32, 34, 37, 44].

Other challenges in teaching were engaging students online, as students turned off the camera and sound, making interactions difficult [33–35, 38, 43]. Online teaching of practical skills was described as more challenging than developing theoretical competence [30, 34, 42]. Moreover, as examinations in theoretical courses shifted from on-campus to an online format [31, 36, 41], studies reported on difficulties assessing students` knowledge and competence [31, 46].

Many studies reported on lack of knowledge on how to use the available online platforms and systems [29, 30, 32, 33, 35, 38, 39, 45], and a need for technical and pedagogical support in the shift to online teaching [29, 31, 35, 41–43]. Some studies describe that institutional support was provided [15, 21, 22, 25], in the form of pedagogical [15, 21, 25], technical [19, 21, 22, 25, 32], as well as emotional, mentoring and administrative support [22]. Support from colleagues was reported to be crucial [28, 32, 44, 46].

Some studies describe positive reactions to changes due to the pandemic. These were self-efficacy in mastering new online platforms [37, 39, 41, 44, 45], and becoming more competent in online teaching skills [29, 43]. Other positive aspects were being challenged [39, 42], and having an opportunity for professional growth [36, 46]. Moreover, studies described online teaching as accessible, flexible and convenient since it did not require travelling [27, 30, 31, 36, 38].

The COVID-19 pandemic’s impact on nursing faculties` research activity received less attention in the included studies. A decrease in publications was measured [27] and a description of challenges with conducting research [36, 45]. One study had research as a main topic and reported that nursing faculty were concerned about the time allocated to teaching and a decline in time for research activity [23].

### Workload and working hours

The professional workload of nursing faculty during the COVID-19 pandemic was described in 14 studies [15, 21, 24, 25, 31, 32, 36–38, 40, 41, 46–48]. Changes in workload were related to the faculty’s increased sense of responsibility to support nursing students online [24, 32, 37, 38, 41, 46, 47]. Other studies measured or described an increase in workload in connection with preparing lectures and teaching and supporting students online [15, 21, 25, 36, 41, 48].

Strategies to manage the workload were presented in two studies. These were sharing resources and collaborating with colleagues to develop course material and preparing for new students earlier than prior to the pandemic [31, 40].

Few studies reported on working hours focusing more on the abrupt change to the online format of courses and attending meetings or workshops [31, 38, 41, 43, 47]. Of these five publications, one described saving travel time [43], whereas others describe strategies of setting time limits for working hours [38].

### Psychological, physical and social aspects

The personal impact of the COVID-19 pandemic on faculty members is included in most of the included publications. Psychological aspects were reported in 24 studies, physical aspects in 7 and social aspects in 12 studies.

The 24 publications that included psychological aspects had measurements or descriptions of stress related to the unknown COVID-19 virus and the pandemics impact on their working conditions [19–21, 24, 26, 38, 44, 47, 48]. Other studies report minimal to mild levels of anxiety and coping well, or moderate levels of anxiety and depression [18, 20]. Furthermore, nursing faculty describe feeling guilty for not working at the frontline with nurses or for the students caring for COVID-19 patients [39–41]. Ten publications describe that nursing faculties mental health deteriorated during the pandemic period with examples of mental and emotional fatigue [17, 36, 40, 41], burnout and decreased emotional well-being [21], feeling overwhelmed, confused and helpless [21, 31, 38, 39, 41, 43, 46], and lacking motivation [36]. Eight studies reported emotional exhaustion or struggle with challenges balancing work and life at home [15, 16, 21, 25, 27, 28, 32, 41]. Strategies used by faculty were reported as prioritizing time for self-care activities [32, 39, 44, 47], slowing down, setting realistic expectations for oneself, and prioritizing [25, 39, 41].

The seven publications that included physical aspects had descriptions of nursing faculty experiencing headache [24, 38, 40], neck pain [40], difficulties with their eyes or vision [24, 36], feeling tired, fatigue [17, 41, 47], and difficulties sleeping [36, 38, 47]. Some of these studies describe nursing faculty trying to cope with strategies to promote their own health e.g., exercise, or developing destructive behavior e.g., gaining weight [38, 41, 47].

The impact of the COVID-19 pandemic on faculty’s social life was reported in 12 studies. This was mainly related to social distance or isolation from colleagues and students. Virtual communication and interaction with colleagues and students was challenging [16, 31, 32], likewise isolation from colleagues [28, 35, 38, 41, 45]. Two studies described the experience of being alone, isolated and missing support from colleagues [35, 38]. Others report the COVID-19 pandemic`s impact on their isolation from family and friends [16, 27, 31, 36]. Strategies for coping with isolation from colleagues were meeting and collaborating with colleagues online [28, 39–41, 44, 46] and by supporting each other by meeting online via Zoom [40, 41, 45].

## Discussion

This study mapped the existing research literature on how the COVID-19 pandemic impacted nursing faculty in academia professionally and personally.

The review revealed that the abrupt shift to online education entailed nursing faculty with significant professional challenges. The included studies reported a pressing need for structured guidance and resources to assist faculty in adapting to this new pedagogical landscape. A review analyzing the methodologies and technologies used in nursing education during the pandemic also reported challenges associated with teaching virtually without being prepared. However, the authors acknowledged the flexibility of virtual class methodologies and the advantage of being able to use synchronous and asynchronous formats [49]. Although new digital tools were learned and taken into use during the pandemic, the digital tools are constantly evolving, which underscores the necessity for ongoing professional development specific to digital teaching modalities. Technology will continue to influence nursing education in the future [50], and incorporating new technologies into teaching and learning processes are among the core competencies of nursing faculty [51]. Two systematic reviews highlighted that digital competence has become a crucial competency for educators today. The reviews also underscored that to effectively implement online learning, nursing curricula will require revisions [52, 53]. Hence, enhancing training in digital teaching methods and technologies is necessary.

Another important finding in this review was that many studies reported that nursing faculty experienced difficulties when transitioning to working from home. The difficulties were connected to the availability of technical resources, and to what degree they experienced administrative, collegial and managerial support. A loss of professional support increased the workload of nursing faculty and adversely affected their work-life balance. These findings align with a narrative study that also identified increased workloads for faculty during the pandemic, and that lack of leadership and administrative support exacerbated these challenges [54]. In our scoping review, we found a few studies that reported on opportunities to conduct research during the COVID-19 pandemic. Research and evidence are important core competencies for nurse educators in developing critical inquiry, conducting research, and use findings in identifying and solving problems in nursing education [51]. Together these findings indicate that many organizations were not sufficiently prepared for the sudden change of employees working from home. Simultaneously, it is well established that organizational changes occur rapidly, and it can be assumed that new situations requiring transitions to home office will occur again. Educational organizations can prepare for such events and thereby reduce occupational stressors and the workload for their employees. This is supported by The International Council of Nurses (ICN), who called on global health leaders and policymakers to support economic growth by investing in education and improving working conditions for all nurses [55]. Based on these findings, it is recommended that educational organizations implement structured support systems for nursing faculty to mitigate the challenges associated with remote working. Specifically, providing systems for remote leadership support, administrative support and remote collegial support is recommended to alleviate frustration related to remote learning responsibilities.

Findings from multiple studies in this review described the emotional and physical difficulties of faculty, and a need for support. A recent meta-synthesis examining faculty’s caring strategies across continents during the COVID-19 pandemic corroborates these findings [56]. Faced with overwhelming work pressure, extended hours, and unsustainable workloads, many faculty members experienced significant emotional and physical exhaustion [57]. According to Dugger (2024), heavy workloads and lack of work-life balance are the most significant contributing factors to burnout and intent to leave nursing academic. This is a particular concern in light of the Global strategic directions for nursing and midwifery (2021–2025) which identify a shortage of qualified faculty to educate at the bachelor`s degree level and above [51].

The pandemic has worsened the existing shortage of nurses, leading to urgent and rapid policy measures aimed at increasing the nurse supply across countries [58]. The ICN encourages implementing physical and emotional well-being strategies for all nurses, including nursing faculty, to address global health challenges [55]. A focus on the long-term impact of the pandemic on nursing faculty in academia is recommended in future studies, and to develop interventions aiming at well-being of faculty in nursing education.

### Methodological considerations

The search strategy was developed in collaboration with an academic librarian and peer reviewed by another academic librarian. The search included eight databases and other resources. We chose a broad search strategy limited to literature from August 2019 onwards, as the COVID-19 virus first appeared during autumn of 2019 aiming for sensitivity instead of specificity [59]. To ensure the retrieval of all relevant publications, studies were included for full text screening if another researcher deemed the study eligible for screening of title and abstract.

In the full text screening, we experienced some challenges related to the term nursing faculty being described in various ways (e.g., faculty, educator, teacher, instructor, lecturer and having different roles across countries (e-g, clinical instructor). Also, the distinction between professional and personal impact was sometimes difficult (e.g., social aspects). Although we cannot ignore the fact that some studies may have been missed, we ensured the inclusion of relevant studies by two reviewers reading the full text independently and solving disagreements through consensus-based discussions with a third reviewer.

Although the protocol for this scoping review has not been registered or published, the researchers have followed the non-published protocol. Moreover, the publications included in this study were not assessed for quality. This is optional according to the methods used in this scoping review [10].

## Conclusions

This scoping review mapped the literature on how the COVID-19 pandemic impacted faculty in nursing education professionally and personally. The educational institutions in nursing were unprepared for such a crisis as the COVID-19 pandemic. The findings describe a pressing need for structured guidance and resources to assist faculty in adapting transitioning to online teaching and working from home. This review describes the personal aspects of the pandemic with emotional and physical difficulties of faculty and a need for support. Implementing physical and emotional well-being strategies for nursing faculty globally is encouraged.

## Electronic supplementary material

Below is the link to the electronic supplementary material.

Supplementary Material 1

Supplementary Material 2

Supplementary Material 3

## Data Availability

The authors confirms that all data generated or analysed during this study are included in this published article.

## Abbreviations

JBI: Joanna Briggs Institute
PRISMA ScR: Preferred Reporting Items for Systematic Reviews and Meta-Analysis Extension for Scoping Reviews
ICN: International Council of Nurses
