# Supplementary material for: The impact of the COVID-19 pandemic on faculty in nursing education: a scoping review
Source: BMC Nurs. 2025 Jul 8;24:880. doi: 10.1186/s12912-025-03550-7 (PMC12235766; doi:10.1186/s12912-025-03550-7)
Supplement: Supplementary file 1 — Supplementary Material 1 [file 12912_2025_3550_MOESM1_ESM.docx]

### Appendix I: Search strategy for Medline

### **Database: Ovid MEDLINE(R) and Epub Ahead of Print, In-Process, In-Data-Review & Other Non-Indexed Citations and Daily <1946 to June 15, 2023>** **Search Strategy:** **1**  Faculty, Nursing/ (10826) **2**  exp Education, Nursing/ (89302) **3**  ((nurse or nursing or nurses) adj (staff or teach* or facult* or educat* or training or practition*)).ti,ab. (54441) **4**  or/1-3 (126742) **5**  exp COVID-19/ (228248) **6**  SARS-CoV-2/ (155105) **7**  (covid-19 or covid next 19).ti,ab. (307181) **8**  Pandemics/ (115443) **9**  exp Coronavirus Infections/ (239797) **10**  ((COVID adj "19") or COVID-19 or COVID19).ti,ab. (308086) **11**  exp Coronavirus/ (168900) **12**  Coronavirus 229E, Human/ (390) **13**  (Coronavirus* or (corona adj virus*)).ti,ab. (123430) **14**  exp Pneumovirus Infections/ (8815) **15**  (Pneumonia adj virus*).ti,ab. (339) **16**  (COVID or NCOV or 2019NCOV or CORONAVIRINAE).ti,ab. (313286) **17**  Severe Acute Respiratory Syndrome/ (5734) **18**  (("19" or "2019") adj2 (epidem* or epidemy or epidemic* or pandem*)).ti,ab. (129862) **19**  (sars cov 2 or sars2 or sarscov2 or sarscov-2 or cov 2019 or sars coronavirus 2 or sars corona virus 2 or sars-cov-2).ti,ab. (104447) **20**  or/5-19 (398152) **21**  4 and 20 (1708)
