# Supplementary material for: The impact of the COVID-19 pandemic on faculty in nursing education: a scoping review
Source: BMC Nurs. 2025 Jul 8;24:880. doi: 10.1186/s12912-025-03550-7 (PMC12235766; doi:10.1186/s12912-025-03550-7)
Supplement: Supplementary file 2 — Supplementary Material 2 [file 12912_2025_3550_MOESM2_ESM.docx]

Documentation on search strategy on the topic COVID-19 and nursing education.

# Medline

Date of search: 20^th^ June 2022

Number of hits (before duplication removal): 649

Comments:

Documentation of search:

Database: Ovid MEDLINE(R) and Epub Ahead of Print, In-Process, In-Data-Review & Other Non-Indexed Citations and Daily <1946 to June 17, 2022>

Search Strategy:

--------------------------------------------------------------------------------

1 exp Faculty, Nursing/ (10580)

2 exp Education, Nursing/ (87738)

3 ((nurse or nursing) adj (school* or college* or teach* or facult* or educat* or training or lecture* or teach* or curriculum or learn* or program* or class*)).ti,ab. (31959)

4 or/1-3 (101185)

5 exp COVID-19/ (168281)

6 exp SARS-CoV-2/ (128891)

7 exp Pandemics/ (88937)

8 exp Coronavirus Infections/ (179288)

9 ((COVID adj "19") or COVID-19 or COVID19).ti,ab. (223589)

10 exp Coronavirus/ (142062)

11 exp Coronavirus 229E, Human/ (332)

12 (Coronavirus* or (corona adj virus*)).ti,ab. (97607)

13 exp Pneumovirus Infections/ (8297)

14 (Pneumonia adj virus*).ti,ab. (335)

15 (COVID or NCOV or 2019NCOV or CORONAVIRINAE).ti,ab. (226999)

16 exp Severe Acute Respiratory Syndrome/ (5692)

17 (("19" or "2019") adj2 (epidem* or epidemy or epidemic* or pandem*)).ti,ab. (90344)

18 (sars cov 2 or sars2 or sarscov2 or sarscov-2 or cov 2019 or sars coronavirus 2 or sars corona virus 2 or sars-cov-2).ti,ab. (75979)

19 or/5-18 (298654)

20 4 and 19 (660)

21 limit 20 to yr="2019 -Current" (649)

***************************

# Embase

Date of search: 20^th^ June 2022

Number of hits (before duplication removal): 685

Comments:

Documentation of search:

Database: Embase <1974 to 2022 Week 24>

Search Strategy:

--------------------------------------------------------------------------------

1 exp nursing education/ (86806)

2 ((nurse or nursing) adj (school* or college* or teach* or facult* or educat* or training or lecture* or teach* or curriculum or learn* or program* or class*)).ti,ab. (32188)

3 or/1-2 (95937)

4 exp coronavirus disease 2019/ (225957)

5 exp Severe acute respiratory syndrome coronavirus 2/ (67121)

6 exp pandemic/ (125851)

7 exp Coronavirus infection/ (245818)

8 ((COVID adj "19") or COVID-19 or COVID19).ti,ab. (239904)

9 exp Coronavirinae/ (89314)

10 exp Human coronavirus 229E/ (656)

11 (Coronavirus* or (corona adj virus*)).ti,ab. (99557)

12 exp Pneumovirus infection/ (6943)

13 (Pneumonia adj virus*).ti,ab. (336)

14 (COVID or NCOV or 2019NCOV or CORONAVIRINAE).ti,ab. (244254)

15 exp severe acute respiratory syndrome/ (10824)

16 (("19" or "2019") adj2 (epidem* or epidemy or epidemic* or pandem*)).ti,ab. (94158)

17 (sars cov 2 or sars2 or sarscov2 or sarscov-2 or cov 2019 or sars coronavirus 2 or sars corona virus 2 or sars-cov-2).ti,ab. (82616)

18 or/4-17 (342610)

19 3 and 18 (706)

20 limit 19 to yr="2019 -Current" (685)

***************************

# Cinahl

Date of search: 20^th^ June 2022

Number of hits (before duplication removal): 1192

Comments:

Documentation of search:

| **#** | **Query** | **Limiters/Expanders** | **Last Run Via** | **Results** |
| --- | --- | --- | --- | --- |
| S1 | (MH "Faculty, Nursing") | Expanders - Apply equivalent subjects  Search modes - Boolean/Phrase | Interface - EBSCOhost Research Databases  Search Screen - Advanced Search  Database - CINAHL | 17,347 |
| S2 | (MH "Education, Nursing+") | Expanders - Apply equivalent subjects  Search modes - Boolean/Phrase | Interface - EBSCOhost Research Databases  Search Screen - Advanced Search  Database - CINAHL | 79,760 |
| S3 | TI ( (nurse or nursing) N0 (school* or college* or teach* or facult* or educat* or training or lecture* or teach* or curriculum or learn* or program* or class*) ) OR AB ( (nurse or nursing) N0 (school* or college* or teach* or facult* or educat* or training or lecture* or teach* or curriculum or learn* or program* or class*) ) | Expanders - Apply equivalent subjects  Search modes - Boolean/Phrase | Interface - EBSCOhost Research Databases  Search Screen - Advanced Search  Database - CINAHL | 51,656 |
| S4 | S1 OR S2 OR S3 | Expanders - Apply equivalent subjects  Search modes - Boolean/Phrase | Interface - EBSCOhost Research Databases  Search Screen - Advanced Search  Database - CINAHL | 113,843 |
| S5 | (MH "COVID-19+") | Expanders - Apply equivalent subjects  Search modes - Boolean/Phrase | Interface - EBSCOhost Research Databases  Search Screen - Advanced Search  Database - CINAHL | 33,594 |
| S6 | (MH "SARS-CoV-2") | Expanders - Apply equivalent subjects  Search modes - Boolean/Phrase | Interface - EBSCOhost Research Databases  Search Screen - Advanced Search  Database - CINAHL | 963 |
| S7 | (MH "Disease Outbreaks+") | Expanders - Apply equivalent subjects  Search modes - Boolean/Phrase | Interface - EBSCOhost Research Databases  Search Screen - Advanced Search  Database - CINAHL | 70,540 |
| S8 | (MH "Coronavirus Infections") | Expanders - Apply equivalent subjects  Search modes - Boolean/Phrase | Interface - EBSCOhost Research Databases  Search Screen - Advanced Search  Database - CINAHL | 2,332 |
| S9 | TI ( (COVID N0 "19") or COVID-19 or COVID19 ) OR AB ( (COVID N0 "19") or COVID-19 or COVID19 ) | Expanders - Apply equivalent subjects  Search modes - Boolean/Phrase | Interface - EBSCOhost Research Databases  Search Screen - Advanced Search  Database - CINAHL | 81,407 |
| S10 | (MH "Coronavirus+") | Expanders - Apply equivalent subjects  Search modes - Boolean/Phrase | Interface - EBSCOhost Research Databases  Search Screen - Advanced Search  Database - CINAHL | 2,715 |
| S11 | TI ( Coronavirus* or (corona N0 virus*) ) OR AB ( Coronavirus* or (corona N0 virus*) ) | Expanders - Apply equivalent subjects  Search modes - Boolean/Phrase | Interface - EBSCOhost Research Databases  Search Screen - Advanced Search  Database - CINAHL | 24,725 |
| S12 | TI Pneumonia N0 virus* OR AB Pneumonia N0 virus* | Expanders - Apply equivalent subjects  Search modes - Boolean/Phrase | Interface - EBSCOhost Research Databases  Search Screen - Advanced Search  Database - CINAHL | 65 |
| S13 | TI ( COVID or NCOV or 2019NCOV or CORONAVIRINAE ) OR AB ( COVID or NCOV or 2019NCOV or CORONAVIRINAE ) | Expanders - Apply equivalent subjects  Search modes - Boolean/Phrase | Interface - EBSCOhost Research Databases  Search Screen - Advanced Search  Database - CINAHL | 86,220 |
| S14 | (MH "Severe Acute Respiratory Syndrome") | Expanders - Apply equivalent subjects  Search modes - Boolean/Phrase | Interface - EBSCOhost Research Databases  Search Screen - Advanced Search  Database - CINAHL | 2,502 |
| S15 | TI ( ("19" or "2019") N2 (epidem* or epidemy or epidemic* or pandem*) ) OR AB ( ("19" or "2019") N2 (epidem* or epidemy or epidemic* or pandem*) ) | Expanders - Apply equivalent subjects  Search modes - Boolean/Phrase | Interface - EBSCOhost Research Databases  Search Screen - Advanced Search  Database - CINAHL | 4,724 |
| S16 | TI ( sars cov 2 or sars2 or sarscov2 or sarscov-2 or cov 2019 or sars coronavirus 2 or sars corona virus 2 or sars-cov-2 ) OR AB ( sars cov 2 or sars2 or sarscov2 or sarscov-2 or cov 2019 or sars coronavirus 2 or sars corona virus 2 or sars-cov-2 ) | Expanders - Apply equivalent subjects  Search modes - Boolean/Phrase | Interface - EBSCOhost Research Databases  Search Screen - Advanced Search  Database - CINAHL | 14,251 |
| S17 | S5 OR S6 OR S7 OR S8 OR S9 OR S10 OR S11 OR S12 OR S13 OR S14 OR S15 OR S16 | Expanders - Apply equivalent subjects  Search modes - Boolean/Phrase | Interface - EBSCOhost Research Databases  Search Screen - Advanced Search  Database - CINAHL | 133,254 |
| S18 | S4 AND S17 | Expanders - Apply equivalent subjects  Search modes - Boolean/Phrase | Interface - EBSCOhost Research Databases  Search Screen - Advanced Search  Database - CINAHL | 1,293 |
| S19 | S4 AND S17 | Limiters - Published Date: 20190101-20221231  Expanders - Apply equivalent subjects  Search modes - Boolean/Phrase | Interface - EBSCOhost Research Databases  Search Screen - Advanced Search  Database - CINAHL | 1,192 |

# Scopus

Date of search: 20^th^ June 2022

Number of hits (before duplication removal): 640

Comments:

Documentation of search:


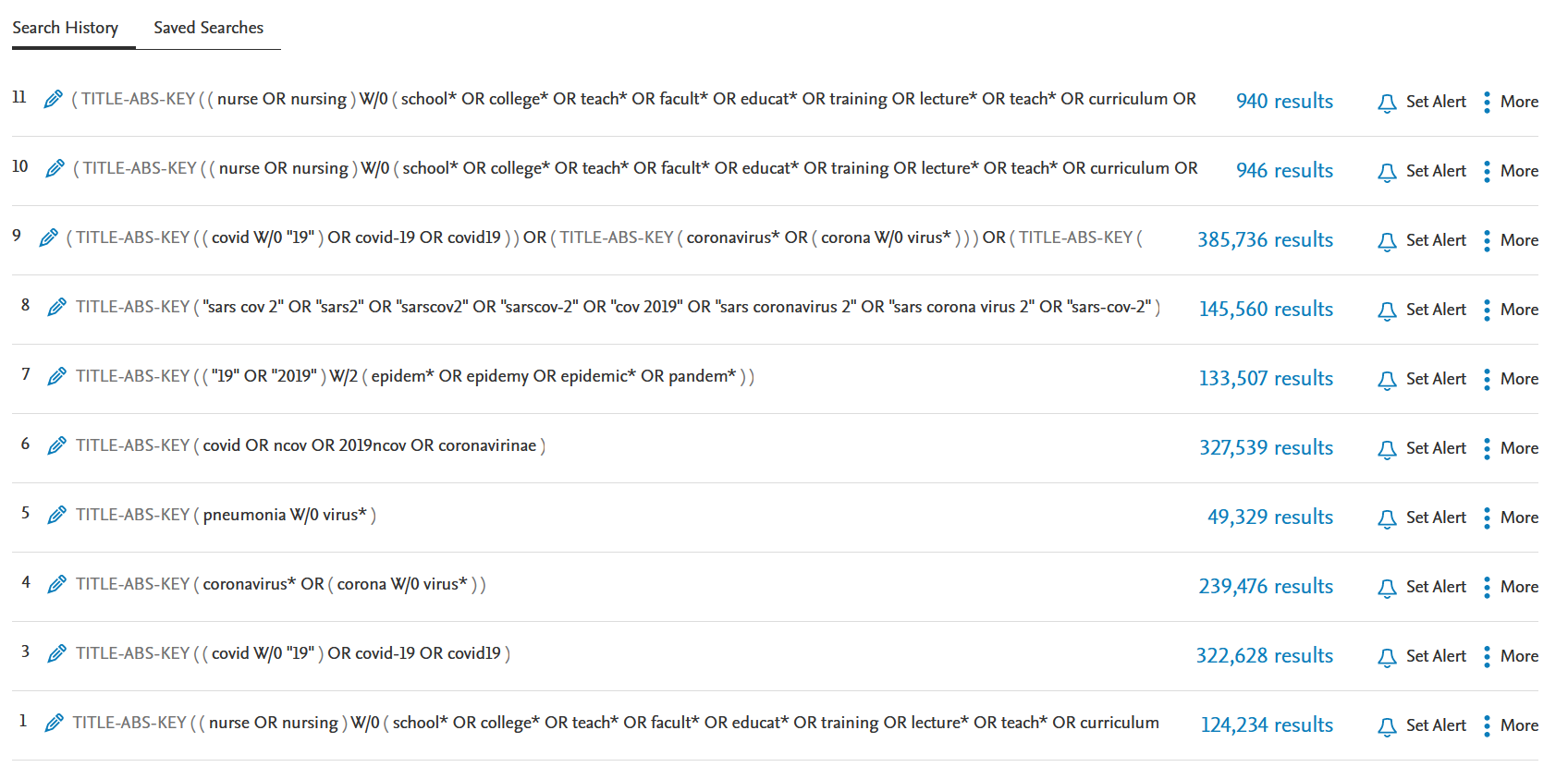


# Web of Science

Date of search: 20^th^ June 2022

Number of hits (before duplication removal): 569

Comments:

Documentation of search:


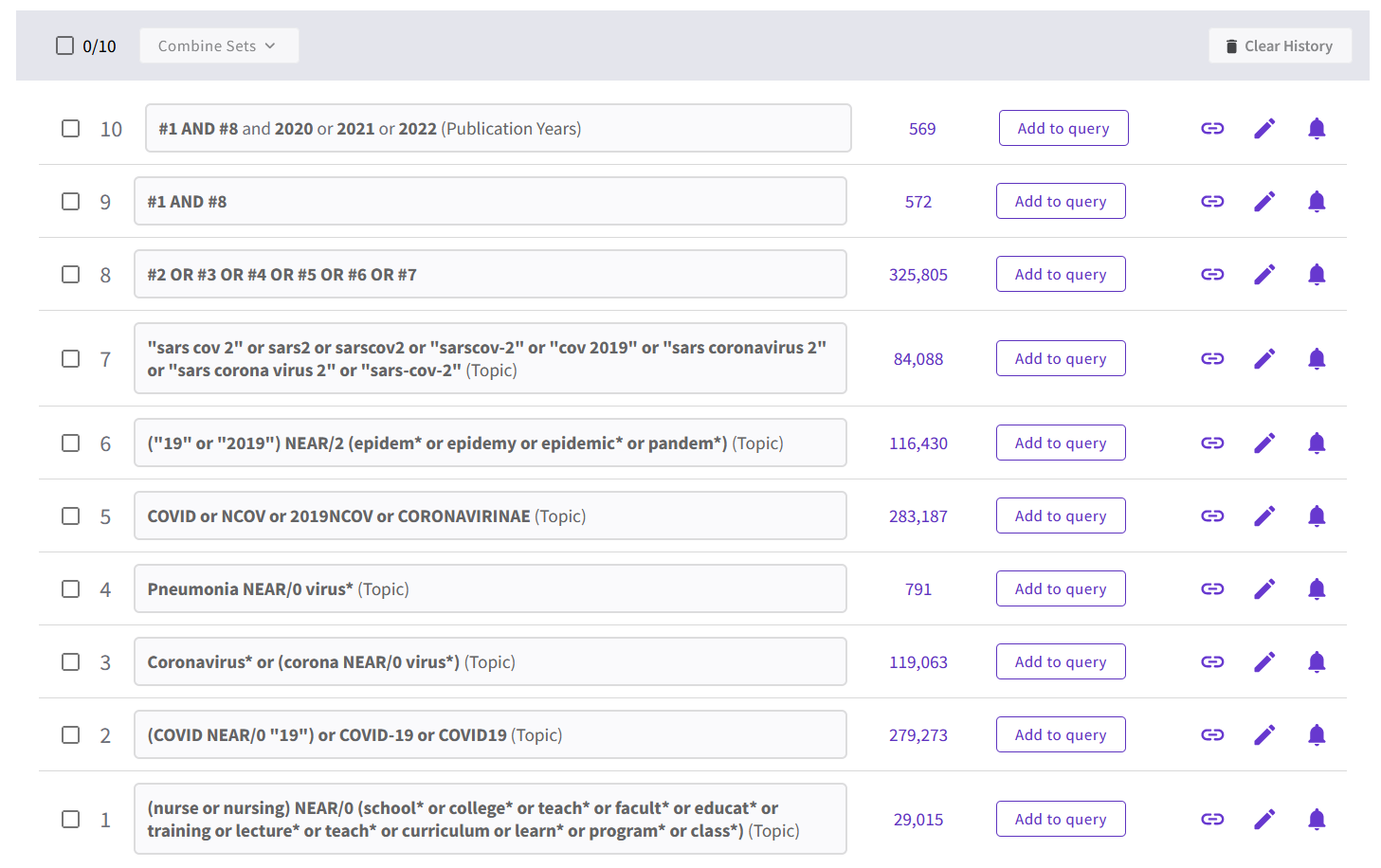


# Cochrane

Date of search: 20^th^ June 2022

Number of hits (before duplication removal): 0 reviews, 5 trials

Comments: Limited the search to publications from 2019 to today.

Documentation of search:


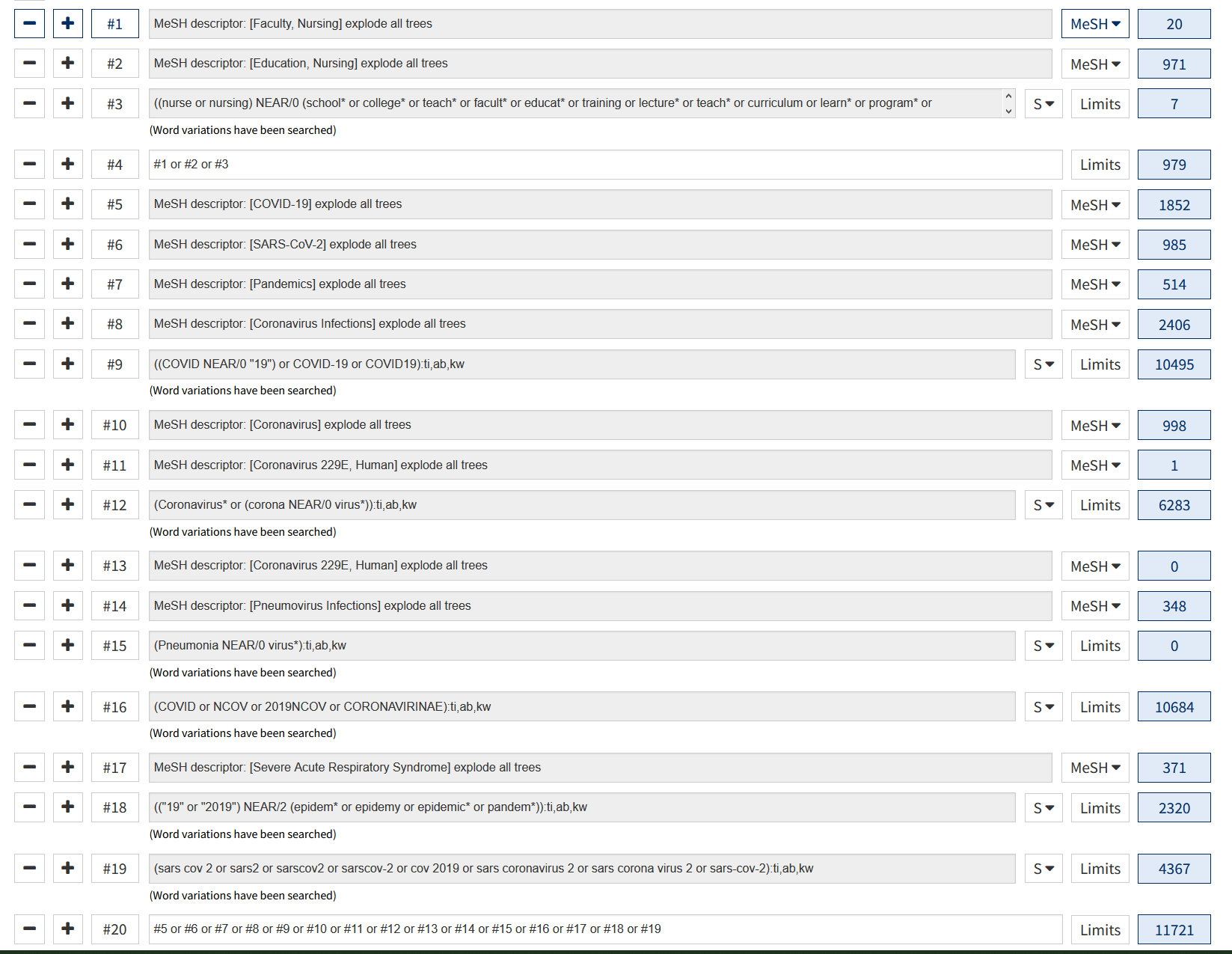


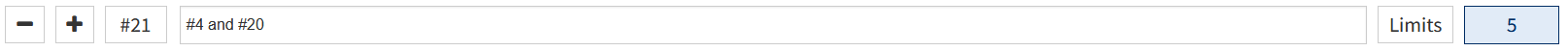


# ERIC

Date of search: 20^th^ June 2022

Number of hits (before duplication removal): 12

Comments:

Documentation of search:

| **#** | **Query** | **Limiters/Expanders** | **Last Run Via** | **Results** |
| --- | --- | --- | --- | --- |
| S1 | TI ( (nurse or nursing) N0 (school* or college* or teach* or facult* or educat* or training or lecture* or teach* or curriculum or learn* or program* or class*) ) OR AB ( (nurse or nursing) N0 (school* or college* or teach* or facult* or educat* or training or lecture* or teach* or curriculum or learn* or program* or class*) ) | Expanders - Apply equivalent subjects  Search modes - Boolean/Phrase | Interface - EBSCOhost Research Databases  Search Screen - Advanced Search  Database - ERIC | 5,028 |
| S2 | DE "COVID-19" OR DE "Pandemics" | Expanders - Apply equivalent subjects  Search modes - Boolean/Phrase | Interface - EBSCOhost Research Databases  Search Screen - Advanced Search  Database - ERIC | 5,046 |
| S3 | TI ( (COVID N0 "19") or COVID-19 or COVID19 ) OR AB ( (COVID N0 "19") or COVID-19 or COVID19 ) | Expanders - Apply equivalent subjects  Search modes - Boolean/Phrase | Interface - EBSCOhost Research Databases  Search Screen - Advanced Search  Database - ERIC | 4,612 |
| S4 | TI ( Coronavirus* or (corona N0 virus*) ) OR AB ( Coronavirus* or (corona N0 virus*) ) | Expanders - Apply equivalent subjects  Search modes - Boolean/Phrase | Interface - EBSCOhost Research Databases  Search Screen - Advanced Search  Database - ERIC | 821 |
| S5 | TI ( COVID or NCOV or 2019NCOV or CORONAVIRINAE ) OR AB ( COVID or NCOV or 2019NCOV or CORONAVIRINAE ) | Expanders - Apply equivalent subjects  Search modes - Boolean/Phrase | Interface - EBSCOhost Research Databases  Search Screen - Advanced Search  Database - ERIC | 4,631 |
| S6 | TI ( ("19" or "2019") N2 (epidem* or epidemy or epidemic* or pandem*) ) OR AB ( ("19" or "2019") N2 (epidem* or epidemy or epidemic* or pandem*) ) | Expanders - Apply equivalent subjects  Search modes - Boolean/Phrase | Interface - EBSCOhost Research Databases  Search Screen - Advanced Search  Database - ERIC | 139 |
| S7 | TI ( "sars cov 2" or sars2 or sarscov2 or "sarscov-2" or "cov 2019" or "sars coronavirus 2" or "sars corona virus 2" or "sars-cov-2" ) OR AB ( "sars cov 2" or sars2 or sarscov2 or "sarscov-2" or "cov 2019" or "sars coronavirus 2" or "sars corona virus 2" or "sars-cov-2" ) | Expanders - Apply equivalent subjects  Search modes - Boolean/Phrase | Interface - EBSCOhost Research Databases  Search Screen - Advanced Search  Database - ERIC | 63 |
| S8 | S2 OR S3 OR S4 OR S5 OR S6 OR S7 | Expanders - Apply equivalent subjects  Search modes - Boolean/Phrase | Interface - EBSCOhost Research Databases  Search Screen - Advanced Search  Database - ERIC | 5,574 |
| S9 | S1 AND S8 | Expanders - Apply equivalent subjects  Search modes - Boolean/Phrase | Interface - EBSCOhost Research Databases  Search Screen - Advanced Search  Database - ERIC | 12 |
| S10 | S1 AND S8 | Limiters - Date Published: 20190101-20211231  Expanders - Apply equivalent subjects  Search modes - Boolean/Phrase | Interface - EBSCOhost Research Databases  Search Screen - Advanced Search  Database - ERIC | 12 |
| S11 | S1 AND S8 | Limiters - Date Published: 20200101-20221231  Expanders - Apply equivalent subjects  Search modes - Boolean/Phrase | Interface - EBSCOhost Research Databases  Search Screen - Advanced Search  Database - ERIC | 12 |

# Teacher Reference Center

Date of search: 20^th^ June 2022

Number of hits (before duplication removal): 7

Comments:

Documentation of search:

| **#** | **Query** | **Limiters/Expanders** | **Last Run Via** | **Results** |
| --- | --- | --- | --- | --- |
| S1 | TI ( (nurse or nursing) N0 (school* or college* or teach* or facult* or educat* or training or lecture* or teach* or curriculum or learn* or program* or class*) ) OR AB ( (nurse or nursing) N0 (school* or college* or teach* or facult* or educat* or training or lecture* or teach* or curriculum or learn* or program* or class*) ) | Expanders - Apply equivalent subjects  Search modes - Boolean/Phrase | Interface - EBSCOhost Research Databases  Search Screen - Advanced Search  Database - Teacher Reference Center | 685 |
| S2 | (ZU "covid-19") or (ZU "covid-19 pandemic") or (ZU "sars-cov-2") or (ZU "pandemics") or (ZU "coronaviruses") | Expanders - Apply equivalent subjects  Search modes - Boolean/Phrase | Interface - EBSCOhost Research Databases  Search Screen - Advanced Search  Database - Teacher Reference Center | 3,515 |
| S3 | TI ( (COVID N0 "19") or COVID-19 or Coronavirus* or (corona N0 virus*) ) OR AB ( (COVID N0 "19") or COVID-19 or Coronavirus* or (corona N0 virus*) ) | Expanders - Apply equivalent subjects  Search modes - Boolean/Phrase | Interface - EBSCOhost Research Databases  Search Screen - Advanced Search  Database - Teacher Reference Center | 3,042 |
| S4 | TI ( COVID or NCOV or 2019NCOV or CORONAVIRINAE or sars cov 2 or sars2 or sarscov2 or sarscov-2 or cov 2019 or sars coronavirus 2 or sars corona virus 2 or sars-cov-2 ) OR AB ( COVID or NCOV or 2019NCOV or CORONAVIRINAE or sars cov 2 or sars2 or sarscov2 or sarscov-2 or cov 2019 or sars coronavirus 2 or sars corona virus 2 or sars-cov-2 ) | Expanders - Apply equivalent subjects  Search modes - Boolean/Phrase | Interface - EBSCOhost Research Databases  Search Screen - Advanced Search  Database - Teacher Reference Center | 2,747 |
| S5 | TI ( ("19" or "2019") N2 (epidem* or epidemy or epidemic* or pandem*) ) OR AB ( ("19" or "2019") N2 (epidem* or epidemy or epidemic* or pandem*) ) | Expanders - Apply equivalent subjects  Search modes - Boolean/Phrase | Interface - EBSCOhost Research Databases  Search Screen - Advanced Search  Database - Teacher Reference Center | 105 |
| S6 | S2 OR S3 OR S4 OR S5 | Expanders - Apply equivalent subjects  Search modes - Boolean/Phrase | Interface - EBSCOhost Research Databases  Search Screen - Advanced Search  Database - Teacher Reference Center | 4,137 |
| S7 | S1 AND S6 | Expanders - Apply equivalent subjects  Search modes - Boolean/Phrase | Interface - EBSCOhost Research Databases  Search Screen - Advanced Search  Database - Teacher Reference Center | 7 |
| S8 | S1 AND S6 | Limiters - Published Date: 20190101-20221231  Expanders - Apply equivalent subjects  Search modes - Boolean/Phrase | Interface - EBSCOhost Research Databases  Search Screen - Advanced Search  Database - Teacher Reference Center | 7 |

# Epistemonikos

Date of search: 20^th^ June 2022

Number of hits (before duplication removal): 77

Comments: I’ve limited the hits to systematic reviews

Documentation of search:

((nurse or nursing) and (school* or college* or teach* or facult* or educat* or training or lecture* or teach* or curriculum or learn* or program* or class*)) and (COVID-19 or COVID19 OR

Coronavirus* or COVID or NCOV or 2019NCOV or CORONAVIRINAE)

# Google Scholar

Date of search: 20^th^ June 2022

Number of hits (before duplication removal): 200

Comments:I’ve only included the first 200 hits, as relevance is decreasing after this.

Documentation of search:

1. covid|corona|coronavirus (nurse|nursing

school|college|faculty|education|training|lecture|curriculum|programme|class)
