# Supplementary material for: The impact of the COVID-19 pandemic on faculty in nursing education: a scoping review
Source: BMC Nurs. 2025 Jul 8;24:880. doi: 10.1186/s12912-025-03550-7 (PMC12235766; doi:10.1186/s12912-025-03550-7)
Supplement: Supplementary file 3 — Supplementary Material 3 [file 12912_2025_3550_MOESM3_ESM.docx]

Documentation on search strategy on the topic COVID-19 and nursing education (updated).

# Medline

Date of search: 19th September 2023

Number of hits (before duplication removal): 614

Comments:

Documentation of search:

**Database: Ovid MEDLINE(R) and Epub Ahead of Print, In-Process, In-Data-Review & Other Non-Indexed Citations and Daily <1946 to September 18, 2023>**
**Search Strategy:**
**1**  exp Faculty, Nursing/ (10870)
**2**  exp Education, Nursing/ (89595)
**3**  ((nurse or nursing) adj (school* or college* or teach* or facult* or educat* or training or lecture* or teach* or curriculum or learn* or program* or class*)).ti,ab. (34111)
**4**  or/1-3 (104266)
**5**  exp COVID-19/ (239702)
**6**  exp SARS-CoV-2/ (159363)
**7**  exp Pandemics/ (120067)
**8**  exp Coronavirus Infections/ (251331)
**9**  ((COVID adj "19") or COVID-19 or COVID19).ti,ab. (323718)
**10**  exp Coronavirus/ (173263)
**11**  exp Coronavirus 229E, Human/ (398)
**12**  (Coronavirus* or (corona adj virus*)).ti,ab. (128023)
**13**  exp Pneumovirus Infections/ (8928)
**14**  (Pneumonia adj virus*).ti,ab. (339)
**15**  (COVID or NCOV or 2019NCOV or CORONAVIRINAE).ti,ab. (329307)
**16**  exp Severe Acute Respiratory Syndrome/ (5735)
**17**  (("19" or "2019") adj2 (epidem* or epidemy or epidemic* or pandem*)).ti,ab. (137306)
**18**  (sars cov 2 or sars2 or sarscov2 or sarscov-2 or cov 2019 or sars coronavirus 2 or sars corona virus 2 or sars-cov-2).ti,ab. (109573)
**19**  or/5-18 (416766)
**20**  4 and 19 (1068)
**21**  limit 20 to yr="2022 -Current" (614)

# Embase

Date of search: 19th September 2023

Number of hits (before duplication removal): 596

Comments:

Documentation of search:

**Database: Embase <1974 to 2023 Week 37>**
**Search Strategy:**
**1**  exp nursing education/ (89465)
**2**  ((nurse or nursing) adj (school* or college* or teach* or facult* or educat* or training or lecture* or teach* or curriculum or learn* or program* or class*)).ti,ab. (34455)
**3**  or/1-2 (99505)
**4**  exp coronavirus disease 2019/ (349511)
**5**  exp severe acute respiratory syndrome/ (11534)
**6**  exp pandemic/ (182260)
**7**  exp Coronavirus infection/ (369677)
**8**  ((COVID adj "19") or COVID-19 or COVID19).ti,ab. (361374)
**9**  exp Coronavirinae/ (124351)
**10**  exp Human coronavirus 229E/ (946)
**11**  (Coronavirus* or (corona adj virus*)).ti,ab. (133292)
**12**  exp Pneumovirus infection/ (8109)
**13**  (Pneumonia adj virus*).ti,ab. (341)
**14**  (COVID or NCOV or 2019NCOV or CORONAVIRINAE).ti,ab. (369788)
**15**  (("19" or "2019") adj2 (epidem* or epidemy or epidemic* or pandem*)).ti,ab. (147239)
**16**  (sars cov 2 or sars2 or sarscov2 or sarscov-2 or cov 2019 or sars coronavirus 2 or sars corona virus 2 or sars-cov-2).ti,ab. (125086)
**17**  or/4-16 (502462)
**18**  3 and 17 (1107)
**19**  limit 18 to yr="2022 -Current" (596)

# Cinahl

Date of search: 19th September 2023

Number of hits (before duplication removal): 953

Comments:

Documentation of search:

| **#** | **Query** | **Results** |
| --- | --- | --- |
| S1 | (MH "Faculty, Nursing") | 17,552 |
| S2 | (MH "Education, Nursing+") | 80,602 |
| S3 | TI ( (nurse or nursing) N0 (school* or college* or teach* or facult* or educat* or training or lecture* or teach* or curriculum or learn* or program* or class*) ) OR AB ( (nurse or nursing) N0 (school* or college* or teach* or facult* or educat* or training or lecture* or teach* or curriculum or learn* or program* or class*) ) | 53,736 |
| S4 | S1 OR S2 OR S3 | 115,641 |
| S5 | (MH "COVID-19+") | 44,101 |
| S6 | (MH "SARS-CoV-2") | 1,443 |
| S7 | (MH "Disease Outbreaks+") | 87,248 |
| S8 | (MH "Coronavirus Infections+") | 48,163 |
| S9 | TI ( (COVID N0 "19") or COVID-19 or COVID19 ) OR AB ( (COVID N0 "19") or COVID-19 or COVID19 ) | 117,594 |
| S10 | (MH "Coronavirus+") | 3,230 |
| S11 | TI ( Coronavirus* or (corona N0 virus*) ) OR AB ( Coronavirus* or (corona N0 virus*) ) | 31,273 |
| S12 | TI Pneumonia N0 virus* OR AB Pneumonia N0 virus* | 72 |
| S13 | TI ( COVID or NCOV or 2019NCOV or CORONAVIRINAE ) OR AB ( COVID or NCOV or 2019NCOV or CORONAVIRINAE ) | 124,706 |
| S14 | (MH "Severe Acute Respiratory Syndrome") | 2,535 |
| S15 | TI ( ("19" or "2019") N2 (epidem* or epidemy or epidemic* or pandem*) ) OR AB ( ("19" or "2019") N2 (epidem* or epidemy or epidemic* or pandem*) ) | 6,408 |
| S16 | TI ( sars cov 2 or sars2 or sarscov2 or sarscov-2 or cov 2019 or sars coronavirus 2 or sars corona virus 2 or sars-cov-2 ) OR AB ( sars cov 2 or sars2 or sarscov2 or sarscov-2 or cov 2019 or sars coronavirus 2 or sars corona virus 2 or sars-cov-2 ) | 19,682 |
| S17 | S5 OR S6 OR S7 OR S8 OR S9 OR S10 OR S11 OR S12 OR S13 OR S14 OR S15 OR S16 | 177,385 |
| S18 | S4 AND S17 | 1,977 |
| S19 | S4 AND S17 | 953 |

# Web of Science

Date of search: 19th September 2023

Number of hits (before duplication removal): 679

Comments:

Documentation of search:


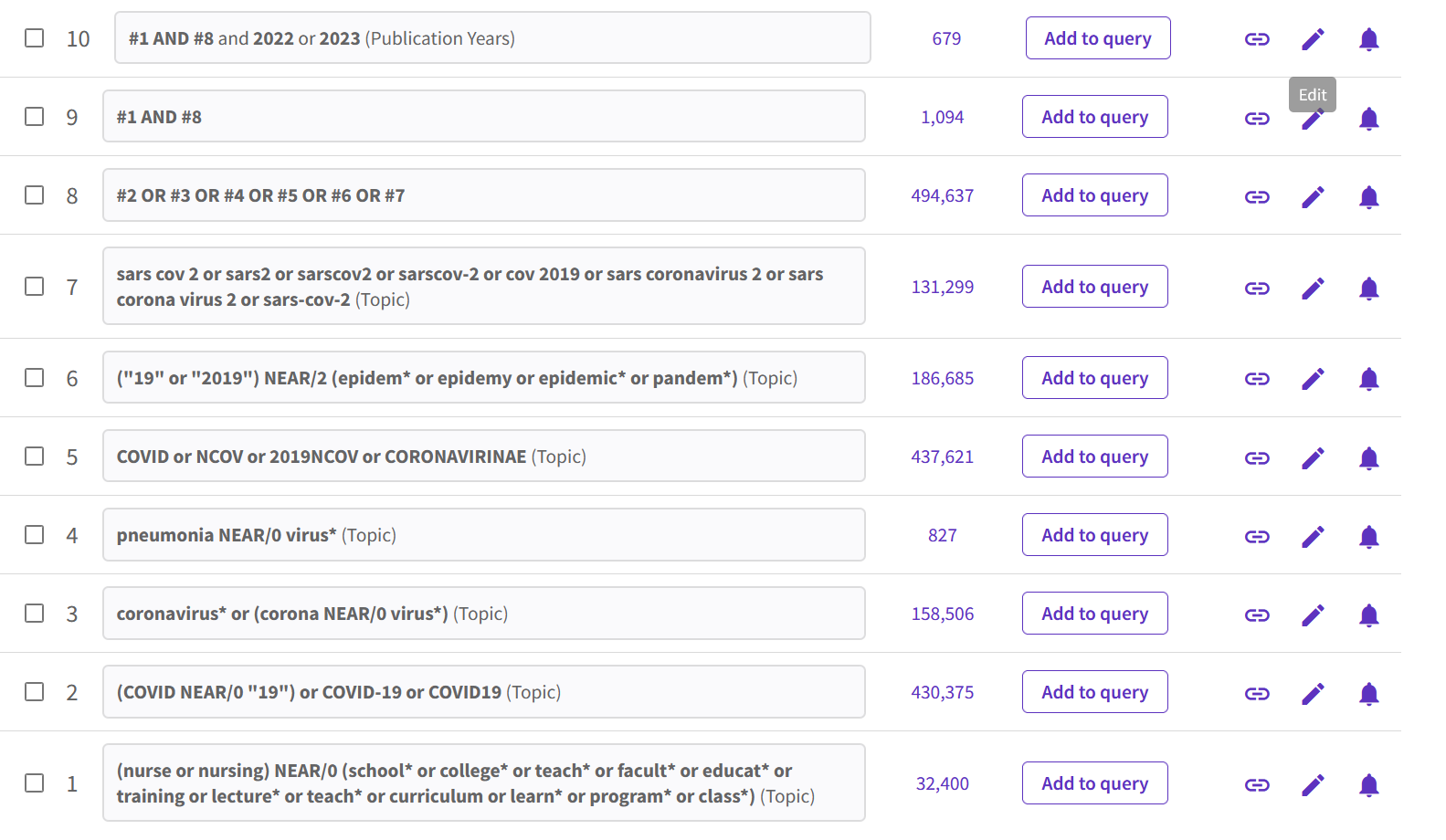


# Cochrane

Date of search: 19th September 2023

Number of hits (before duplication removal): 0 reviews, 29 trials

Comments:

Documentation of search:

ID Search Hits

#1 MeSH descriptor: [Faculty, Nursing] explode all trees 29

#2 MeSH descriptor: [Education, Nursing] explode all trees 1147

#3 ((nurse or nursing) NEXT (school* or college* or teach* or facult* or educat* or training or lecture* or teach* or curriculum or learn* or program* or class*)):ti,ab,kw (Word variations have been searched) 2468

#4 #1 or #2 or #3 2897

#5 MeSH descriptor: [COVID-19] explode all trees 4737

#6 MeSH descriptor: [SARS-CoV-2] explode all trees 2376

#7 MeSH descriptor: [Pandemics] explode all trees 1494

#8 MeSH descriptor: [Coronavirus Infections] explode all trees 5328

#9 ((COVID NEXT "19") or COVID-19 or COVID19):ti,ab,kw (Word variations have been searched) 16722

#10 MeSH descriptor: [Coronavirus] explode all trees 2406

#11 MeSH descriptor: [Coronavirus 229E, Human] explode all trees 1

#12 (coronavirus* or (corona NEXT virus*)):ti,ab,kw (Word variations have been searched) 10536

#13 MeSH descriptor: [Pneumovirus Infections] explode all trees 459

#14 (pneumonia NEXT virus*):ti,ab,kw (Word variations have been searched) 16

#15 (COVID or NCOV or 2019NCOV or CORONAVIRINAE):ti,ab,kw (Word variations have been searched) 17107

#16 MeSH descriptor: [Severe Acute Respiratory Syndrome] explode all trees 389

#17 (("19" or "2019") NEAR/2 (epidem* or epidemy or epidemic* or pandem*)):ti,ab,kw (Word variations have been searched) 4261

#18 (sars cov 2 or sars2 or sarscov2 or sarscov-2 or cov 2019 or sars coronavirus 2 or sars corona virus 2 or sars-cov-2):ti,ab,kw (Word variations have been searched) 6727

#19 #5 OR #6 OR #7 OR #8 OR #9 OR #10 OR #11 OR #12 OR #13 OR #14 OR #15 OR #16 OR #17 OR #18 18761

#20 #4 and #19 29

# ERIC

Date of search: 19th September 2023

Number of hits (before duplication removal): 26

Comments:

Documentation of search:

| **#** | **Query** | **Results** |
| --- | --- | --- |
| S1 | TI ( (nurse or nursing) N0 (school* or college* or teach* or facult* or educat* or training or lecture* or teach* or curriculum or learn* or program* or class*) ) OR AB ( (nurse or nursing) N0 (school* or college* or teach* or facult* or educat* or training or lecture* or teach* or curriculum or learn* or program* or class*) ) OR KW ( (nurse or nursing) N0 (school* or college* or teach* or facult* or educat* or training or lecture* or teach* or curriculum or learn* or program* or class*) ) | 5,228 |
| S2 | DE "COVID-19" OR DE "Pandemics" | 12,855 |
| S3 | TI ( (COVID N0 "19") or COVID-19 or COVID19 ) OR AB ( (COVID N0 "19") or COVID-19 or COVID19 ) OR KW ( (COVID N0 "19") or COVID-19 or COVID19 ) | 11,914 |
| S4 | TI ( Coronavirus* or (corona N0 virus*) ) OR AB ( Coronavirus* or (corona N0 virus*) ) OR KW ( Coronavirus* or (corona N0 virus*) ) | 1,599 |
| S5 | TI ( COVID or NCOV or 2019NCOV or CORONAVIRINAE ) OR AB ( COVID or NCOV or 2019NCOV or CORONAVIRINAE ) OR KW ( COVID or NCOV or 2019NCOV or CORONAVIRINAE ) | 11,954 |
| S6 | TI ( ("19" or "2019") N2 (epidem* or epidemy or epidemic* or pandem*) ) OR AB ( ("19" or "2019") N2 (epidem* or epidemy or epidemic* or pandem*) ) OR KW ( ("19" or "2019") N2 (epidem* or epidemy or epidemic* or pandem*) ) | 329 |
| S7 | TI ( "sars cov 2" or sars2 or sarscov2 or "sarscov-2" or "cov 2019" or "sars coronavirus 2" or "sars corona virus 2" or "sars-cov-2" ) OR AB ( "sars cov 2" or sars2 or sarscov2 or "sarscov-2" or "cov 2019" or "sars coronavirus 2" or "sars corona virus 2" or "sars-cov-2" ) OR KW ( "sars cov 2" or sars2 or sarscov2 or "sarscov-2" or "cov 2019" or "sars coronavirus 2" or "sars corona virus 2" or "sars-cov-2" ) | 136 |
| S8 | S2 OR S3 OR S4 OR S5 OR S6 OR S7 | 14,464 |
| S9 | S1 AND S8 | 42 |
| S10 | S1 AND S8 | 26 |

# Teacher Reference Center

Date of search: 19th September 2023

Number of hits (before duplication removal): 2

Comments:

Documentation of search:

| **#** | **Query** | **Results** |
| --- | --- | --- |
| S1 | TI ( (nurse or nursing) N0 (school* or college* or teach* or facult* or educat* or training or lecture* or teach* or curriculum or learn* or program* or class*) ) OR AB ( (nurse or nursing) N0 (school* or college* or teach* or facult* or educat* or training or lecture* or teach* or curriculum or learn* or program* or class*) ) | 701 |
| S2 | (ZU "covid-19") or (ZU "covid-19 pandemic") or (ZU "sars-cov-2") or (ZU "pandemics") or (ZU "coronaviruses") | 4,507 |
| S3 | TI ( (COVID N0 "19") or COVID-19 or Coronavirus* or (corona N0 virus*) ) OR AB ( (COVID N0 "19") or COVID-19 or Coronavirus* or (corona N0 virus*) ) | 4,052 |
| S4 | TI ( COVID or NCOV or 2019NCOV or CORONAVIRINAE or sars cov 2 or sars2 or sarscov2 or sarscov-2 or cov 2019 or sars coronavirus 2 or sars corona virus 2 or sars-cov-2 ) OR AB ( COVID or NCOV or 2019NCOV or CORONAVIRINAE or sars cov 2 or sars2 or sarscov2 or sarscov-2 or cov 2019 or sars coronavirus 2 or sars corona virus 2 or sars-cov-2 ) | 3,726 |
| S5 | TI ( ("19" or "2019") N2 (epidem* or epidemy or epidemic* or pandem*) ) OR AB ( ("19" or "2019") N2 (epidem* or epidemy or epidemic* or pandem*) ) | 137 |
| S6 | S2 OR S3 OR S4 OR S5 | 5,388 |
| S7 | S1 AND S6 | 8 |
| S8 | S1 AND S6 | 2 |

# Epistemonikos

Date of search: 19th September 2023

Number of hits (before duplication removal): 104

Comments: I’ve only included systematic reviews.

Documentation of search:

((nurse or nursing) and (school* or college* or teach* or facult* or educat* or training or lecture* or teach* or curriculum or learn* or program* or class*)) and (COVID-19 or COVID19 OR Coronavirus* or COVID or NCOV or 2019NCOV or CORONAVIRINAE)

# Scopus

Date of search: 19th September 2023

Number of hits (before duplication removal): 986

Comments:

Documentation of search:


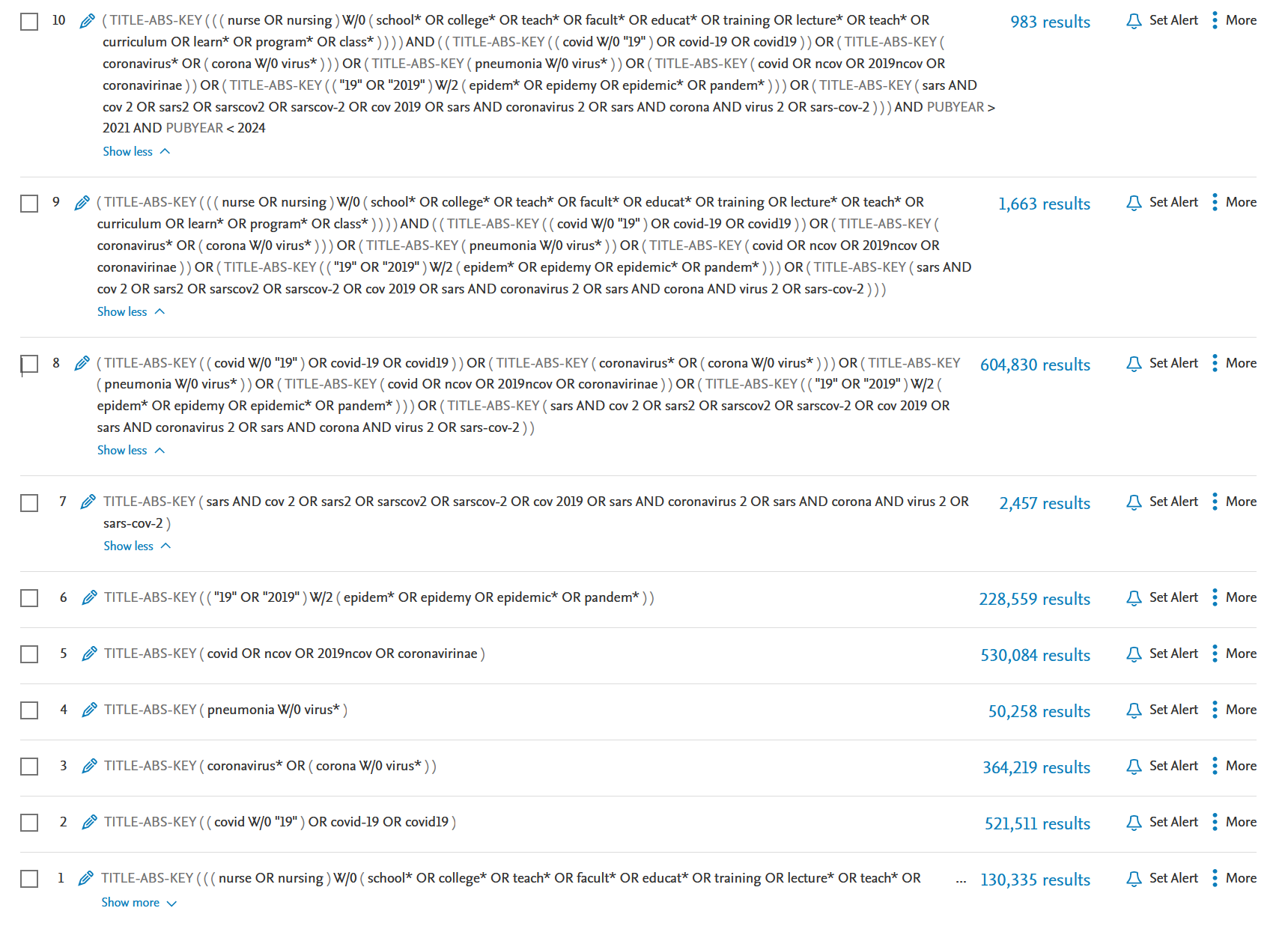


# Google Scholar

Date of search: 19th September 2023

Number of hits (before duplication removal): 200

Comments:I’ve only imported the first 200 hits. This is because relevance is usually reduced after the the first 200 hits.

Documentation of search:

1. covid|corona|coronavirus (nurse|nursing

school|college|faculty|education|training|lecture|curriculum|programme|class)
